# Is endometrial thickness associated with fertility outcomes in intrauterine insemination? a cohort study

**DOI:** 10.3389/fendo.2025.1705695

**Published:** 2026-01-09

**Authors:** Tong Du, Wentao Li, Suqun Zhang, Qifeng Lyu, Yanping Kuang, Ben W. Mol, Yun Wang, Xiaoyan Mao, Dong Zhao

**Affiliations:** 1 Department of Assisted Reproduction, Shanghai Ninth People’s Hospital, Shanghai Jiao Tong University School of Medicine, Shanghai, China; 2 Department of Obstetrics and Gynecology, The School of Clinical Sciences at Monash Health, Monash University, Clayton, VIC, Australia; 3 Aberdeen Centre for Women’s Health Research, School of Medicine, Medical Sciences and Nutrition, University of Aberdeen, Aberdeen, United Kingdom; 4 Department of Obstetrics and Gynecology, Shanghai Ninth People’s Hospital, Shanghai Jiao Tong University School of Medicine, Shanghai, China

**Keywords:** endometrial thickness, intrauterine insemination, clinical pregnancy, live birth, fertility outcome

## Abstract

**Background:**

Intrauterine insemination (IUI) is the first line treatment for conditions such as unexplained or mild male factor infertility. Endometrial thickness (EMT) is an important indicator for predicting pregnancy outcomes in *in-vitro* fertilization. However, published data about whether EMT has a predictive capacity for success in IUI is controversial, and most studies suggest that endometrial thickness is not associated with IUI success, which limits its use in IUI.

**Methods:**

This was a single center retrospective cohort study that included women undergoing IUI cycles from January 2007 to June 2021. We categorized EMT into thin (<7 mm), medium (7–14 mm), and thick (>14 mm) groups. For all IUI cycles, we computed adjusted odds ratios (aORs) and 95% confidence intervals (CIs) using Generalized Estimating Equation Regression Models. For first IUI cycles specifically, we applied both Inverse Propensity Score Weighted Regression Adjustment Models and Propensity Score Matching Analyses to compare fertility outcomes. Moreover, we computed the predicted probability of primary outcomes for continuous EMT in mm using restricted cubic splines, allowing for non-linear relationships.

**Results:**

This cohort included 13103 IUI cycles involving 7609 women. Across all cycles, live birth rates were lower in the thin EMT group (11.0%) and higher in the thick EMT group (16.9%), compared to the medium EMT group (13.5%)—aOR 0.82 (95% CI 0.67-0.998) for thin EMT and aOR 1.22 (95% CI 1.02-1.45) for thick EMT. The results were consistent when analyzing first cycles only. Restricted cubic spline analysis revealed a linear positive gradient that suggests a progressive increase in live birth rates with increasing EMT.

**Conclusions:**

In natural or Letrozole with or without Human Menopausal Gonadotropin stimulated IUI cycles, EMT on trigger day is a significant predictor of live birth, with thin EMT associated with reduced success rates. EMT measurements could serve as a useful marker in IUI treatment.

## Background

Endometrium, embryo, and the synchrony of the two are essential components of a pregnancy. Endometrium thickness (EMT), which can usually be conveniently measured by transvaginal ultrasound, is the most commonly used indicator of endometrium quality in obstetrics and gynecology.

Intrauterine insemination (IUI) is the first line treatment for conditions such as unexplained and mild male factor infertility and is one of the most used methods of assisted reproduction worldwide (1).

However, published data about EMT’s predictive capacity for clinical pregnancy after IUI are controversial (**Table 1**) (2–16). Counterintuitively, most studies found no evidence for an association between EMT and clinical pregnancy rates following IUI, and suggest that canceling IUI cycles because of a thin endometrium may negatively affect clinical care (2–11). Only a few studies have observed a significant increase in pregnancy rates with increasing EMT, suggesting that targeted therapies to optimize EMT could represent a promising avenue for improving fertility success rates in IUI (14–16).

**Table 1:** Summary of selected previous studies on the predictive value of endometrial thickness in IUI.

Study	Region	Design	Period	Indication	OS Protocol	EMT measure day	N		Study interval (mm)			Primary outcome	Follicle adjusting	Predictive value
							Women	Cycles						
Hock et al.1997 (2) *Fertil Steril*	USA	Prospective Single center	1992.06-1993.12	NA	CC+hMG	hCG trigger day	223	223	Per mm			PR	No. of >15 mm follicles	No
Kolibianakis et al.2003 (3) *RBM Online*	Belgium	Prospective Single center	2001.05-2002.05	None severe male factor; dysovulation; lesbian couples; unexplained	CC	hCG trigger day	168	168	Per mm	<6		OPR	No	No
										6-<8				
										≥8				
Asadi et al.2014 (4) *Arch Gynecol Obstet*	Iran	Prospective Single center	2011.09-2012.02	PCOS	CC+hMG	IUI day (66 h after hCG trigger)	110	110	Per mm			OPR	No. of dominant follicles	No
Weiss et al.2017^a^ (5) *Hum Reprod*	Multi-	Meta-Analysis	NA- 2013.02	Unexplained; unexplained + mild male factor	uFSH/rFSH; CC/CC+ hMG; LE	hCG trigger day; IUI day; LH surge day; cycle day 10-11-12	1525 in total (105-446 each)	1525 in total (105-446 each)	Per mm			CPR LBR	No	No
Liu et al.2019 (6) *Reprod Biol Endocrin*	Canada	Retrospective Single center	2012.01-2015.12	Unexplained; PCOS; hypothalamic hypogonadism	rFSH; rFSH+hMG	Within 36h of LH surge or hCG trigger	548	1065	< 7			CPR	No. of ≥15 mm follicles	No
									7–10.4					
									10.5–13.9					
									≥14					
Danhof et al. 2020 (7) *Hum Reprod Open*	Nether-lands	Prospective Multicenter	2013.07-2016.03	Unexplained	rFSH/uFSH/hMG; CC	hCG trigger day	666	1968	Per mm			OPR	No. of follicles: 2 vs. 1, or 3 vs. 1 (No size information)	No
Li et al.2020 (8) *J Int* *Med Res*	China	Retrospective Single center	2015.01-2019.06	Unexplained; PCOS	Gn	hCG trigger day	361	930	Per mm			CPR	No. of ≥14 mm follicles	No
Quaas et al.2021(9) *Fertil Steril*	USA	Prospective 12 centers	NA	Unexplained	Gn; CC; LE	hCG trigger day	868	2459	≤5			LBR	No. of ≥16 mm follicles	No
									6–8					
									9–12					
									≥13					
Rachmawati et al.2023 (10) *BMC* *Res Notes*	Indonesia	Retrospective 2 centers	2021.12- NA	Unexplained	CC; LE	NA	122	NA	<8			BPR	Dominant follicle size: 18–22 vs. ≥22 mm	No
									8–10					
									≥10					
Zhang et al.2024(11) *Reprod Biol*	USA	Retrospective Single center	2017.03-2023.03	Excluding uterine factor	Gn; CC; LE	hCG trigger day	NA	2281	< 7			CPR LBR	No	No
									≥7					
Vagios et al.2023 (12) *J Assist Reprod Genet*	USA	Retrospective Single center	2007.11- 2020.03	Excluding uterine factor	Gn	hCG trigger day or the day before	964	1016 (idiopathic infertility: NA)	Per mm			CPR LBR	No	No, except for couples with idiopathic infertility
Lu et al.2024 (13) *Front Endocrinol*	USA	Retrospective Single center	2004.01- 2021.09	Excluding uterine factor or severe tubal/ peritoneal factor with untreated hydrosalpinges.	rFSH; CC	The last ultrasound evaluation	Gn: 556 CC: 877	Gn: 1307 CC: 1938	Per mm	Gn:	CC:	CPR OPR	No	Gn: No CC: Yes
										≤6.9	≤5.7			
										7.0-8.2	5.8-6.9			
										8.3-9.9	7.0-8.1			
										≥10.0	≥8.2			
Dickey et al. 1993 (14) *Fertil Steril*	USA	Prospective Single center	1990.04- 1991.10	Male factor not requiring donor insemination; donor cryopreserved sperm; hostile cervical mucus that did not respond to medical treatment	CC; hMG; CC+hMG; natural cycle	hCG trigger day	271	474	<6			CPR OPR	No	Yes
									6-8					
									≥9					
Wolff et al.2013 (15) *Fertil Steril*	USA	Retrospective Single center	2004– 2011	Unexplained	CC+FSH	hCG trigger day	2929	2929	Per mm			CPR	No. of ≥14 mm follicles	Yes
Tang et al.2024 (16) *Int J Gen Med*	China	Retrospective Single center	2014.03- 2023.06	NA	natural cycle (regular- menses) ; CC/HMG/ FSH (irregular- menses)	Endometrial transform day	NA	1464	<8			CPR	No	<8: Yes >13: No
									8–13					
									>13					

Note: IUI, intrauterine insemination; OS, ovarian stimulation; EMT, endometrial thickness; NA, not available; CC, clomiphene citrate; hMG, human menopausal gonadotropin; hCG, human chorionic gonadotropin; PR, pregnancy rate; OPR, ongoing pregnancy rate; PCOS, polycystic ovary syndrome; uFSH, urinary follicle stimulating hormone; rFSH, recombinant follicle stimulating hormone; LE, letrozole; LH, luteinizing hormone; CPR, clinical pregnancy rate; LBR, live birth rate; BPR, biochemical pregnancy rate; Gn, gonadotropin.

^a^ Included studies in this Meta-Analysis were not shown in this table.

It is important to note that all the above studies had limited sample sizes, and most of them did not account for critical confounders especially the size and number of follicles on the trigger day, which are indicative of the embryonic factor that contributes to success in IUI (17). This lack of control for embryonic factors hampers an independent and robust evaluation of the EMT’s influence on clinical pregnancy after IUI. In addition, other fertility outcomes beyond clinical pregnancy were rarely reported in the literature.

A lack of clear evidence-based guidance can lead to difficulties in clinical decision-making, leaving the couples vulnerable to compromised outcomes of IUI, or the time and financial loss of unnecessary cycle cancellation. Thus, the objective of the current study is to investigate the association of EMT on pregnancy chances and other fertility outcomes in IUI.

## Methods

### Study design and population

This was a retrospective cohort study based on data of infertile couples who underwent IUI using the male partner’s sperm from January 2007 to June 2021. For these couples, the women had at least one patent fallopian tube as determined by hysterosalpingography or laparoscopy, and all the male partners had a count of ≥10 million motile sperm in the ejaculate. Only natural cycles or stimulated cycles using Letrozole or Letrozole combined with Human Menopausal Gonadotropin (hMG) were included. Patients lost to follow-up were not used. In consideration that the sample size of the study was relatively large and missing data were minimal [female body mass index: 1.63% (218/13400), EMT on trigger day: 1.01% (136/13400), follicle number on trigger day: 0.55% (74/13400), female age: 0.03% (4/13400), paternal age: 0.03% (4/13400), year of treatment: 0.03% (4/13400), and in total: 2.22% (297/13400)], listwise deletion was applied in the main analyses, and an additional sensitivity analysis using Multiple Imputation had further confirmed robustness to missing data methods.

### Cycle monitoring and ovarian stimulation

The routine cycle monitoring and ovarian stimulation protocols were similar as published studies (17, 18). In brief, as follows:

In natural cycles, defined as cycles that do not use ovarian stimulation drugs, cycle monitoring begins from menstrual cycle day 10 onwards. Transvaginal ultrasound measurement of follicular number and diameters and serum hormone analysis were applied to monitor the development of ovarian follicles. If the dominant follicle was 14-17mm on menstrual cycle day 10, and the serum luteinizing hormone (LH) and progesterone were both around the basic level, the next monitor was arranged 2 days (dominant follicle of 14-15mm) or 1 day (dominant follicle of 16-17mm) later. If the dominant follicle was <14mm, longer intervals would be scheduled based on the roughly estimated follicular growth rate of 2mm per day and the target follicle size was 18–20 mm.

In stimulated cycles, defined as cycles that use ovarian stimulation drugs, the most used protocol was as follows: oral administration of Letrozole (Jiangsu Hengrui Medicine Co., China) 2.5–5mg per day was initiated from menstrual cycle day 3 to 7, and cycle monitoring was started since menstrual cycle day 10 onwards as in natural cycles. If the leading follicle’s diameter was <14mm on menstrual cycle day 10, 75IU hMG (Anhui Fengyuan Pharmaceutical Co., China) was additionally intramuscular injected daily for a variable duration depending on response, while 75 IU hMG or no stimulation drug was applied if the dominant follicle was ≥14mm on menstrual cycle day 10.

In both natural or stimulated cycles, when at least one mature follicle’s diameter was ≥18mm, along with LH level <20IU/l, progesterone <1.2ng/ml, 5000IU Human Chorionic Gonadotropin (hCG, Lizhu Pharmaceutical Trading Co., China) or 0.1mg triptorelin (Decapeptyl, Ferring Pharmaceuticals) or both were administered to induce ovulation, and IUI was performed about 36h later. In cases where spontaneous LH surges appeared (LH ≥20IU/l or progesterone ≥1.2ng/ml), IUI was performed about 24h later, either with an immediate triggering through methods described above or without a triggering. The triggering drug administering day or spontaneous LH surges appearing day was defined as “trigger day”. The selection of IUI triggering protocol is at the discretion of doctors.

Luteal support was not routinely used, but if symptoms of threatened miscarriage such as vaginal bleeding occurred, 10mg dydrogesterone (Duphaston; Abbott Biologicals, Chicago, IL) was orally applied three times per day.

### Semen preparation and insemination

The routine semen preparation and intra-uterine insemination protocols were as below:

Semen samples were collected through masturbation and processed within 1h after ejaculation. After full liquefaction, the spermatozoa were washed via density gradient centrifugation (360g, 15 min) by Isolate (Irvine Scientific, USA) first. Then they were washed using modified THF Medium (Irvine Scientific, USA, with 10% (v/v) Serum Substitute Supplement, Irvine Scientific) via centrifugation (360g, 3 min). Lastly, the purified spermatozoa were resuspended in THF Medium with a final volume of 0.2ml, which was used for intra-uterine insemination. In each cycle, only one insemination procedure was performed through a soft catheter (Cook Group, USA).

### Exposure and outcome assessment

The primary exposure was EMT on trigger day. The measurement is of the thickest echogenic area from one stratum basalis endometrial interface across the endometrial canal to the other stratum basalis interface in the sagittal plane by highly trained sonographers, using a transvaginal probe (19). Thin endometrium is defined as endometrial thickness <7 mm, while thick endometrium is defined as endometrial thickness >14 mm, as most studies reported (19, 20). These thresholds were pre-specified prior to data analysis.

The primary outcomes were clinical pregnancy rates and live birth rates. Secondary outcomes included biochemical pregnancy loss, multiple pregnancy, ectopic pregnancy, and miscarriage. Outcomes were evaluated for all cycles and first cycles, with the primary focus on all cycles.

Serum β-hCG level was routinely measured 17 days after insemination. For women with positive results in serum β-hCG tests, vaginal ultrasonography was routinely performed 31 days after insemination.

Fertility outcomes were defined based on the International Committee for Monitoring Assisted Reproductive Technology and the World Health Organization revised glossary of ART terminology 2009 (21). Specifically, biochemical pregnancy loss was defined as a pregnancy diagnosed only by positive serum β-hCG but does not develop into a clinical pregnancy. Clinical pregnancy was defined as a pregnancy diagnosed by ultrasonographic visualization of at least one gestational sac or definitive clinical signs of pregnancy, and ectopic pregnancy was included. Ectopic pregnancy was defined as at least one extrauterine cavity gestational sac with a yolk sac confirmed by ultrasonography or verified by histologic evidence of chorionic villi collected from a surgery, irrespective of with or without an intrauterine cavity gestational sac (22). The miscarriage rate was calculated as the total number of induced and spontaneous miscarriages, expressed per 100 IUI cycles. Multiple gestation was defined as more than one intrauterine gestational sacs seen on ultrasound. Live birth rate was calculated as the number of deliveries that resulted in ≥1 live-born baby divided by the number of IUI cycles.

### Analysis and statistics

Patient and treatment characteristics were presented as categorical data and described as frequencies and percentages. Comparisons were made using the chi-square test or Fisher’s exact test as appropriate.

In total, three sets of main analyses were performed. In the first two sets of analysis, we divided EMT into thin (<7mm), medium (7-14mm), and thick (>14mm) groups. First, for all cycles, we used the Generalized Estimated Equations, accounting for the correlation between cycles from the same woman, to compute unadjusted and adjusted odds ratios (aORs) and 95% confidence intervals (CIs) for the associations of fertility outcomes in relation to EMT groups on trigger day. Second, in first cycles only, we used both Inverse Propensity Score Weighted Regression Adjustment Models and Propensity Score Matching Analysis to compute the association between EMT groups and fertility outcomes. Third, to facilitate clinical decision-making and avoid using arbitrary cut-offs, we estimated the predicted probability of clinical pregnancy and live birth for continuous EMT in mm at trigger. Specifically, we used restricted cubic splines with four knots in Multivariable Logistic Regression Models (for first cycles analyses) or Generalized Estimated Equation Regression Models (for all cycles analyses). This approach accommodates potential non-linear relationships between EMT and pregnancy outcomes, which can be visually inspected.

All sets of analyses adjusted for potential confounders including maternal characteristics (female age, body mass index, infertility duration, gravidity, previous preterm birth, previous full-term birth, previous miscarriage, previous ectopic pregnancy, smoking history, and polycystic ovarian syndrome, diagnosed according to the Rotterdam consensus (23)), paternal characteristics (paternal age and mild male factor, defined as with ≥1 abnormal parameter: sperm concentration <15×10^6^/ml, motility <32%, normal morphology <4%, but had at least a total of 10 million motile sperm count), and IUI characteristics (including treatment protocol, diameter and number of follicles on trigger day, and year of treatment). Propensity scores were also calculated based on the above confounders using logistic regression. Matches without replacement were performed using propensity scores through the nearest neighbor random matching algorithm. The matched ratios for thin group vs. medium group, and thick group vs. medium group were both 1:5.

In addition, in consideration of the relatively small sample sizes of thin and thick EMT groups compared with medium EMT group, we performed a sensitivity analysis using a wider grouping interval for thin (<8mm) and thick (>11mm) EMT groups to test the stability and reliability of the results in the main analyses by using Generalized Estimation Equation Regression Models and Logistic Regression Models to repeat the analyses in all cycles and first cycles of IUI, respectively. Also, to assess the generalizability of the results across different treatment protocols (natural cycles and Letrozole with or without hMG cycles), we performed a subgroup analysis in all cycles using Generalized Estimation Equation Regression Models.

Statistical analyses were carried out by using SPSS version 23.0 software (SPSS Inc., Chicago, USA) and Stata Version 17.0 (Stata Corp., College Station, TX, USA).

## Results

**Figure 1:**
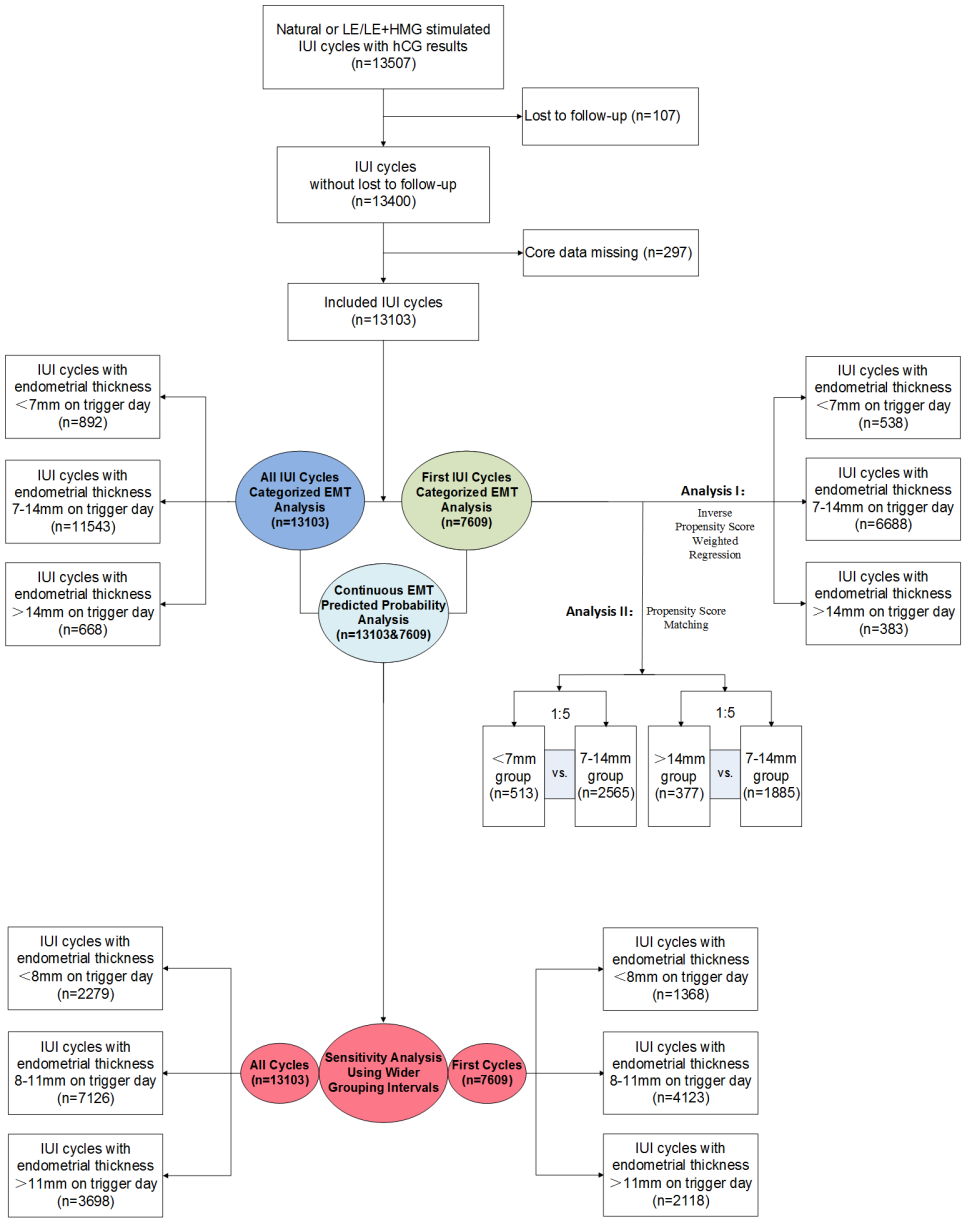
Flowchart of the study.

A total of 13103 IUI cycles from 7609 women were included in this study (**Figure 1**). Of these, 892 IUI cycles had an EMT<7mm on trigger day, 11543 IUI cycles had an EMT of 7-14mm, and 668 IUI cycles had an EMT>14mm. Maternal characteristics including age, infertility duration, gravidity, number of previous miscarriages, and history of ectopic pregnancy, PCOS, paternal age, as well as IUI characteristics, including the number of follicles of different sizes on trigger day and the year of treatment, were significantly differently distributed among the three EMT groups (**Table 2**).

**Table 2:** All cycle analysis: Characteristics of the three endometrial thickness groups in all cycles.

Variable	Thin group (<7 mm)	Medium group (7-14 mm)	Thick group (>14 mm)	*P*
Total cycles, n	892	11543	668	
Maternal characteristics, %				
Female age (y)				**<0.001**
<30	28.5	31.8	34.3	
30-34	43.6	46.0	48.4	
35-37	15.9	14.4	11.1	
38-40	8.5	5.6	4.9	
41-43	2.0	1.6	0.9	
≥44	1.5	0.7	0.4	
Body mass index (kg/m^2^)				0.097
<18.5	13.8	12.0	10.2	
18.5-23.9	60.4	63.6	64.4	
24-28	20.4	17.8	18.6	
>28	5.4	6.6	6.9	
Infertility duration (y)				**<0.001**
<1	16.3	13.4	9.7	
1-2	41.8	38.8	37.9	
3-4	27.4	29.4	29.6	
5-6	9.1	11.7	14.1	
≥7	5.5	6.7	8.7	
Gravidity				**<0.001**
0	54.8	69.9	79.0	
1	24.4	19.2	13.9	
≥2	20.7	11.0	7.0	
Previous preterm birth				0.957
0	99.8	99.7	99.7	
≥1	0.2	0.3	0.3	
Previous full-term birth				0.052
0	90.6	93.0	94.0	
1	9.0	6.6	5.8	
2	0.4	0.4	0.1	
Previous miscarriage				**<0.001**
0	56.4	71.9	81.9	
1	25.2	19.2	12.9	
≥2	18.4	8.9	5.2	
Previous ectopic pregnancy				**0.022**
0	98.3	99.1	99.6	
≥1	1.7	0.9	0.4	
Smoking history				0.392
Yes	0.2	0.1	0.0	
No	99.8	99.9	100.0	
PCOS				**0.018**
Yes	17.8	15.1	12.7	
No	82.2	84.9	87.3	
Paternal characteristics, %				
Paternal age (y)				**<0.001**
<30	19.6	21.3	23.1	
30-39	64.1	68.4	66.5	
40-49	14.5	9.6	9.6	
≥50	1.8	0.7	0.9	
Mild male factor				0.199
Yes	31.8	34.7	35.5	
No	68.2	65.3	64.5	
IUI characteristics, %				
Treatment protocol				0.322
Letrozole stimulation	95.3	94.3	93.6	
Natural cycle	4.7	5.7	6.4	
Follicle number on trigger day				
10-12 mm				0.917
0	80.2	81.1	81.0	
1	13.3	12.3	12.1	
≥2	6.5	6.6	6.9	
12-14 mm				0.096
0	80.9	80.5	77.4	
1	14.3	13.6	14.8	
≥2	4.7	5.9	7.8	
14-16 mm				**<0.001**
0	82.4	77.9	70.8	
1	13.8	16.3	17.7	
≥2	3.8	5.8	11.5	
16-18 mm				**<0.001**
0	76.1	69.5	62.7	
1	21.1	23.8	27.7	
≥2	2.8	6.6	9.6	
18-20 mm				**<0.001**
0	54.1	52.9	51.8	
1	40.8	39.9	35.9	
≥2	5.0	7.2	12.3	
>20 mm				0.544
0	56.4	57.2	56.0	
1	40.4	38.8	39.2	
≥2	3.3	4.0	4.8	
Year of treatment				**<0.001**
2007-2009	2.2	2.1	5.7	
2010-2012	6.7	9.0	15.0	
2013-2015	23.4	27.2	26.5	
2016-2018	40.9	40.2	34.9	
2019-2021	26.7	21.4	18.0	

PCOS, polycystic ovary syndrome; Comparisons were made using chi-square test or Fisher’s exact test as appropriate. Bold indicates significant *P*-values.

### All cycles analysis: generalized estimating equation analysis

In the univariable analysis of all cycles, rates of clinical pregnancy (13.8% vs. 16.5% vs. 20.4%), multiple gestation (1.1% vs. 1.8% vs. 3.3%) and live birth (11.0% vs. 13.5% vs. 16.9%) showed a gradual increase across the thin EMT group, medium EMT group and thick EMT group, respectively. Rates of biochemical pregnancy loss were lowest in the medium EMT group (1.2% vs. 0.5% vs. 1.1%). Rates of ectopic pregnancy and miscarriage were not significantly different among the three EMT groups (**Table 3**).

**Table 3:** All cycle analysis: fertility outcomes of the three endometrial thickness groups in all cycles using Generalized Estimating Equation.

Outcome	Rate, % (n/N)	Crude OR (95% CI)	Adjusted OR^a^ (95% CI)
Biochemical pregnancy loss			
<7 mm	1.2 (11/892)	**2.74 (1.43-5.24)**	**2.55 (1.26-5.17)**
7-14 mm	0.5 (52/11543)	1 (Reference)	1 (Reference)
>14 mm	1.1 (7/668)	**2.33 (1.06-5.12)**	**2.54 (1.13-5.73)**
Clinical pregnancy			
<7 mm	13.8 (123/892)	**0.83 (0.70-0.99)**	**0.83 (0.69-0.98)**
7-14 mm	16.5 (1908/11543)	1 (Reference)	1 (Reference)
>14 mm	20.4 (136/668)	**1.23 (1.05-1.44)**	**1.19 (1.02-1.39)**
Multiple gestation			
<7 mm	1.1 (10/892)	0.64 (0.34-1.20)	0.71 (0.38-1.35)
7-14 mm	1.8 (203/11543)	1 (Reference)	1 (Reference)
>14 mm	3.3 (22/668)	**1.87 (1.21-2.89)**	**1.56 (1.02-2.38)**
Ectopic pregnancy			
<7 mm	0.7 (6/892)	1.58 (0.68-3.69)	1.41 (0.58-3.43)
7-14 mm	0.4 (49/11543)	1 (Reference)	1 (Reference)
>14 mm	0.2 (1/668)	0.35 (0.05-2.55)	0.35 (0.05-2.56)
Miscarriage			
<7 mm	2.2 (20/892)	0.82 (0.53-1.28)	0.78 (0.49-1.23)
7-14 mm	2.7 (315/11543)	1 (Reference)	1 (Reference)
>14 mm	3.3 (22/668)	1.21 (0.79-1.84)	1.09 (0.71-1.67)
Live birth			
<7 mm	11.0 (98/892)	**0.81 (0.67-0.99)**	**0.82 (0.67-0.998)**
7-14 mm	13.5 (1562/11543)	1 (Reference)	1 (Reference)
>14 mm	16.9 (113/668)	**1.25 (1.05-1.49)**	**1.22 (1.02-1.45)**

OR=odds ratio; CI=confidence interval.

a Analyzed using Generalized Estimating Equation for correlated cycles of the same woman, adjusted for female age, paternal age, body mass index, infertility duration, gravidity, previous preterm birth, previous full-term birth, previous miscarriage, previous ectopic pregnancy, smoking, PCOS, mild male factor, treatment protocol, year of treatment, and follicle number and diameter on trigger day; Bold indicates significant *P*-values.

In the adjusted analysis, with the medium EMT group as the reference, chances of clinical pregnancy (aOR 0.83, 95% CI 0.69-0.98 for thin EMT group, aOR 1.19, 95% CI 1.02-1.39 for thick EMT group), multiple gestation (aOR 0.71, 95% CI 0.38-1.35 for thin EMT group, aOR 1.56, 95% CI 1.02-2.38 for thick EMT group), and live birth (aOR 0.82, 95% CI 0.67-0.998 for thin EMT group, aOR 1.22, 95% CI 1.02-1.45 for thick EMT group) also showed a gradual increase across the thin EMT group, medium EMT group and thick EMT group. The differences in biochemical pregnancy loss, ectopic pregnancy, and miscarriage were not significantly different among the three EMT groups (**Table 3**).

### First cycle analysis I: inverse propensity score weighted regression adjustment analysis

In line with the all cycles analysis, in the unweighted first IUI cycles only (n=7609), the rates of clinical pregnancy (14.5% vs. 18.5% vs. 20.1%) and live birth (11.7% vs. 15.2% vs. 17.2%) showed a gradual increase across the thin EMT group, medium EMT group and thick EMT group, respectively. The weighting was satisfactory (**Supplementary Tables 1, 2**). In the weighted cohort, adjusted odds ratios indicated significantly lower odds in the thin EMT group (aOR 0.74, 95% CI 0.54-0.95 for clinical pregnancy; aOR 0.72, 95% CI 0.51-0.93 for live birth) compared to the medium EMT group. Although the odds were higher in the thick EMT group (aOR 1.06, 95% CI 0.77-1.36 for clinical pregnancy; aOR 1.01, 95% CI 0.72-1.30 for live birth), the differences between the thick EMT group and medium EMT group were not statistically significant (**Table 4**).

**Table 4:** First cycle analysis: fertility outcomes of endometrial thickness groups in the first cycles using inverse propensity score weighted regression adjustment.

Outcome	Thin endometrium (<7mm) vs. medium endometrium (7-14mm)			Thick endometrium (>14mm) vs. medium endometrium (7-14mm)		
	Rate in unweighted cohort,% (n/N)	RD in weighted cohort^a^ (95% CI)	OR in weighted cohort^a^ (95% CI)	Rate in unweighted cohort,% (n/N)	RD in weighted cohort^b^ (95% CI)	OR in weighted cohort^b^ (95% CI)
Biochemical pregnancy loss	0.9 (5/538) vs. 0.5 (35/6688)	0.41 (-0.39 to 1.22)	1.78 (0.18-3.38)	1.3 (5/383) vs. 0.5 (35/6688)	0.82 (-0.39 to 2.03)	2.62 (0.09-5.14)
Clinical pregnancy	14.5 (78/538) vs. 18.5 (1234/6688)	**-4.03 (-7.44 to -0.61)**	**0.74 (0.54-0.95)**	20.1 (77/383) vs. 18.5 (1234/6688)	0.93 (-3.40 to 5.27)	1.06 (0.77-1.36)
Multiple gestation	1.5 (8/538) vs. 2.0 (135/6688)	0.04 (-1.32 to 1.40)	1.02 (0.32-1.71)	3.9 (15/383) vs. 2.0 (135/6688)	0.40 (-0.97 to 1.77)	1.20 (0.50-1.91)
Ectopic pregnancy	0.9 (5/538) vs. 0.5 (30/6688)	0.10 (-0.41 to 0.62)	1.23 (0.05-2.42)	0.0 (0/383) vs. 0.5 (30/6688)	**-0.43 (-0.59 to -0.28)**	/
Miscarriage	2.0 (11/538) vs. 3.0 (203/6688)	-0.36 (-2.11 to 1.39)	0.88 (0.30-1.46)	2.9 (11/3383) vs. 3.0 (203/6688)	1.08 (-1.45 to 3.62)	1.37 (0.49-2.25)
Live birth	11.7 (63/538) vs. 15.2 (1013/6688)	**-3.73 (-6.73 to -0.73)**	**0.72 (0.51-0.93)**	17.2 (66/383) vs. 15.2 (1013/6688)	0.10 (-3.63 to 3.83)	1.01 (0.72-1.30)

OR=odds ratio; CI=confidence interval; RD=risk difference.

a Analyzed using Inverse Propensity Score Weighted Regression Adjustment, controlled for female age, paternal age, body mass index, infertility duration, gravidity, previous full-term birth, previous miscarriage, previous ectopic pregnancy, smoking, PCOS, mild male factor, year of treatment, and follicle number and diameter on trigger day; Bold indicates significant *P*-values.

b Same method as the analysis for thin endometrium (<7mm) vs. medium endometrium (7-14mm), except that smoking was dropped because of collinearity.

### First cycle analysis II: propensity score matching analysis

**Figure 2:**
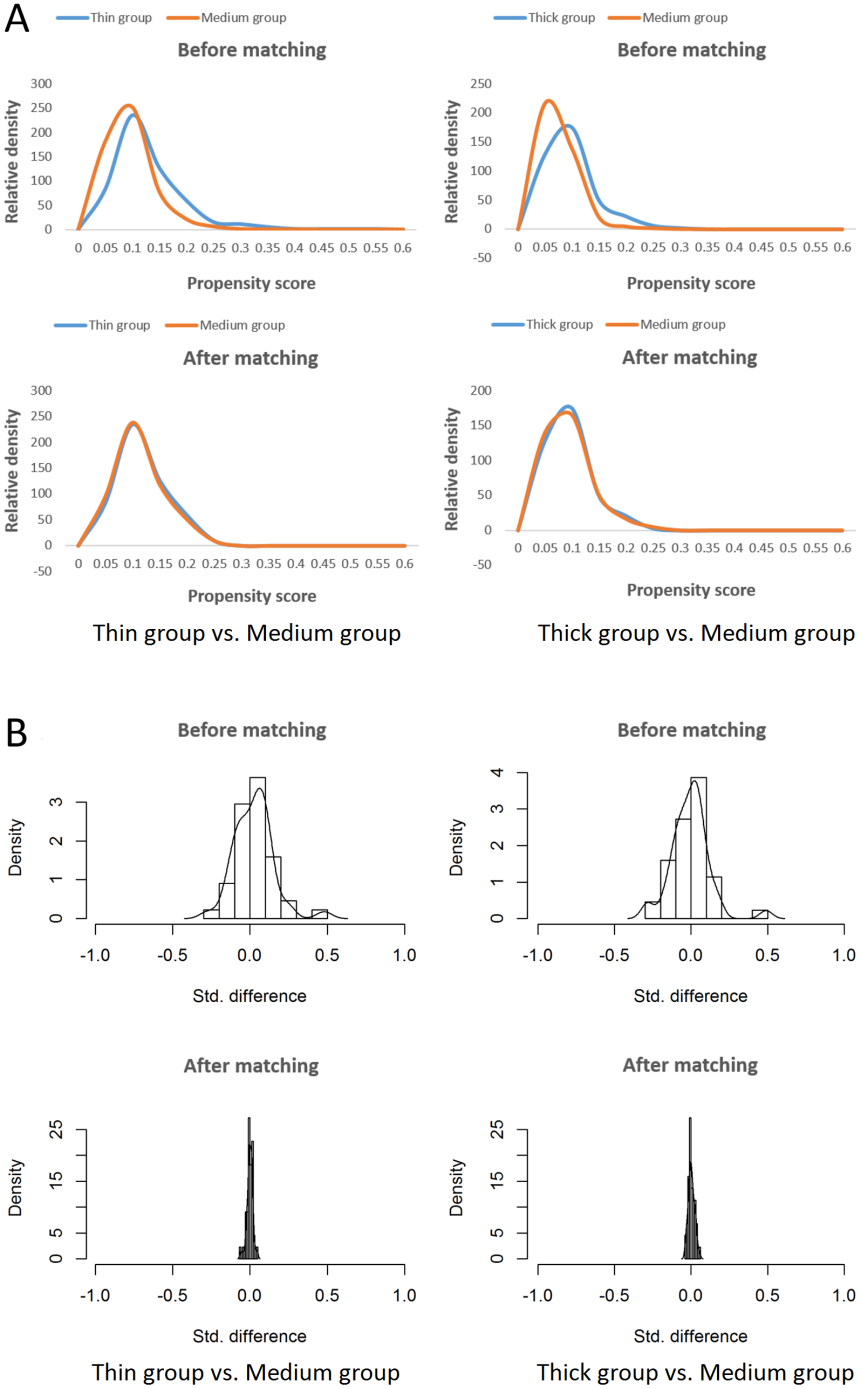
Propensity score matching for thin (<7mm) vs. medium (7–14 mm) EMT cohorts and thick (>14 mm) vs. medium (7–14 mm) EMT cohorts. The distributions of the propensity score **(A)** and standard difference **(B)** indicated balance between the compared cohorts.

In the Propensity Score Matching Analysis, 513 thin EMT women were matched with 2565 women with medium EMT, and 377 women with thick EMT were matched with 1885 women with medium EMT (**Figure 1**). The distributions of propensity scores and standard deviations before and after matching are shown in **Figure 2**. Characteristics of the matched thin, medium, and thick EMT groups are shown in **Table 5**. The distributions of propensity scores, standard differences, and characteristics after matching indicated balance between the comparing cohorts.

**Table 5:** First cycle analysis: characteristics of the endometrial thickness groups in the first cycles after Propensity Score Matching.

Variable	Thin group (<7 mm)	Medium group (7-14 mm)	*P*	Thick group (>14 mm)	Medium group (7-14 mm)	*P*
Total cycles, n	513	2565		377	1885	
Maternal characteristics, %						
Female age (y)			0.995			0.981
<30	31.2	31.4		35.5	34.2	
30-34	44.4	44.8		47.5	47.3	
35-37	14.6	14.9		10.6	11.7	
38-40	6.8	6.4		4.5	5.0	
41-43	1.8	1.5		1.1	1.0	
≥44	1.2	1.1		0.8	0.9	
Body mass index (kg/m^2^)			0.999			0.970
<18.5	13.1	12.9		9.0	9.2	
18.5-23.9	61.0	61.4		64.7	65.6	
24-28	19.5	19.3		18.6	17.5	
>28	6.4	6.4		7.7	7.7	
Infertility duration (y)			1.000			0.968
<1	15.6	15.4		8.8	8.9	
1-2	43.3	43.2		38.2	38.4	
3-4	27.7	27.7		30.8	29.6	
5-6	8.6	8.8		15.1	15.0	
≥7	4.9	4.9		7.2	8.2	
Gravidity			0.887			0.996
0	56.3	57.5		79.6	79.4	
1	25.0	24.3		14.9	15.0	
≥2	18.7	18.2		5.6	5.6	
Previous preterm birth			0.527			1.000
0	100.0	99.9		99.5	99.5	
≥1	0.0	0.1		0.5	0.5	
Previous full-term birth			0.930			0.696
0	91.0	91.3		95.2	94.1	
1	8.4	8.2		4.5	5.6	
2	0.6	0.5		0.3	0.3	
Previous miscarriage			0.832			0.993
0	57.9	59.3		82.5	82.7	
1	26.1	25.2		13.5	13.4	
≥2	16.0	15.5		4.0	3.9	
Previous ectopic pregnancy			0.948			0.841
0	98.4	98.5		99.7	99.8	
≥1	1.6	1.5		0.3	0.2	
Smoking history			0.527			0.655
Yes	0.0	0.1		0.0	0.1	
No	100.0	99.9		100.0	99.9	
PCOS			0.871			0.760
Yes	19.7	19.4		12.7	13.3	
No	80.3	80.6		87.3	86.7	
Paternal characteristics, %						
Paternal age (y)			0.943			0.993
<30	22.0	21.6		22.3	21.7	
30-39	64.9	65.4		67.9	68.5	
40-49	12.5	12.2		9.5	9.5	
≥50	0.6	0.8		0.3	0.3	
Mild male factor			0.677			0.754
Yes	30.8	31.7		36.9	36.0	
No	69.2	68.3		63.1	64.0	
IUI characteristics, %						
Treatment protocol			0.622			0.694
Letrozole stimulation	96.3	96.7		92.0	92.6	
Natural cycle	3.7	3.3		8.0	7.4	
Follicle number on trigger day						
10-12 mm			0.776			0.876
0	77.4	78.8		81.2	81.1	
1	15.0	14.0		11.7	12.3	
≥2	7.6	7.2		7.2	6.6	
12-14 mm			0.974			0.686
0	79.9	80.4		76.1	76.0	
1	15.2	14.9		15.9	17.1	
≥2	4.9	4.7		8.0	6.9	
14-16 mm			0.967			0.844
0	81.9	81.8		71.1	72.5	
1	14.4	14.3		17.5	16.8	
≥2	3.7	3.9		11.4	10.7	
16-18 mm			0.507			0.835
0	75.8	74.5		60.7	62.3	
1	21.4	21.8		30.0	28.5	
≥2	2.7	3.7		9.3	9.1	
18-20 mm			0.605			0.832
0	56.1	54.0		53.6	55.2	
1	39.0	40.3		34.7	33.9	
≥2	4.9	5.7		11.7	10.9	
>20 mm			0.907			0.753
0	55.2	56.2		57.6	57.8	
1	41.3	40.3		36.9	37.5	
≥2	3.5	3.5		5.6	4.7	
Year of treatment			0.994			0.986
2007-2009	1.9	2.1		6.4	5.7	
2010-2012	7.0	7.0		15.6	15.3	
2013-2015	22.8	23.7		26.8	27.5	
2016-2018	42.3	41.6		31.0	31.1	
2019-2021	25.9	25.7		20.2	20.4	

PCOS, polycystic ovary syndrome; Comparisons were made using chi-square test or Fisher’s exact test as appropriate.

As for fertility outcomes, in accordance with the Inverse Propensity Score Weighted Regression Adjustment analysis, the rates of clinical pregnancy (14.6% [75/513] vs. 18.7% [480/2565], OR 0.74, 95% CI 0.57-0.97, *p* = 0.028) and live birth (11.7% [60/513] vs. 15.3% [392/2565], OR 0.73, 95% CI 0.55-0.98, *p* = 0.036) were significantly lower in the thin EMT group compared with medium EMT group. Although the rates were higher in the thick EMT group (20.2% [76/377] vs. 18.3% [345/1885], OR 1.13, 95% CI 0.85-1.49, *p* = 0.398 for clinical pregnancy; 17.2% [65/377] vs. 14.2% [267/1885], OR 1.26, 95% CI 0.94-1.70, *p* = 0.123 for live birth), the differences between the thick EMT group and medium EMT group were not statistically significant (**Table 6**).

**Table 6:** First cycle analysis: Fertility outcomes of endometrial thickness groups in the first cycles after Propensity Score Matching.

Outcome, % (n/N)	Thin group (<7 mm)	Medium group (7-14 mm)	OR	*P*	Thick group (>14 mm)	Medium group (7-14 mm)	OR	*P*
Biochemical pregnancy loss	1.0%(5/513)	0.7%(18/2565)	1.39 (0.52-3.77)	0.512	1.3%(5/377)	0.3%(6/1885)	**4.21 (1.28-13.86)**	**0.010**
Clinical pregnancy	14.6%(75/513)	18.7%(480/2565)	**0.74 (0.57-0.97)**	**0.028**	20.2%(76/377)	18.3%(345/1885)	1.13 (0.85-1.49)	0.398
Multiple gestation	1.4%(7/513)	1.9%(48/2565)	0.73 (0.33-1.61)	0.429	4.0%(15/377)	2.2%(41/1885)	**1.86 (1.02-3.40)**	**0.040**
Ectopic pregnancy	1.0%(5/513)	0.4%(10/2565)	2.52 (0.86-7.39)	0.083	0.0%(0/377)	0.3%(5/1885)	/	0.317
Miscarriage	2.1%(11/513)	3.1%(80/2565)	0.68 (0.36-1.29)	0.234	2.9%(11/377)	4.1%(78/1885)	0.70 (0.37-1.32)	0.266
Live birth	11.7%(60/513)	15.3%(392/2565)	**0.73 (0.55-0.98)**	**0.036**	17.2%(65/377)	14.2%(267/1885)	1.26 (0.94-1.70)	0.123

Comparisons were made using two-sided chi-square test or Fisher’s exact test as appropriate. Bold indicates significant *P*-values.

### Predicted probability of clinical pregnancy and live birth for continuous EMT in mm

**Figure 3:**
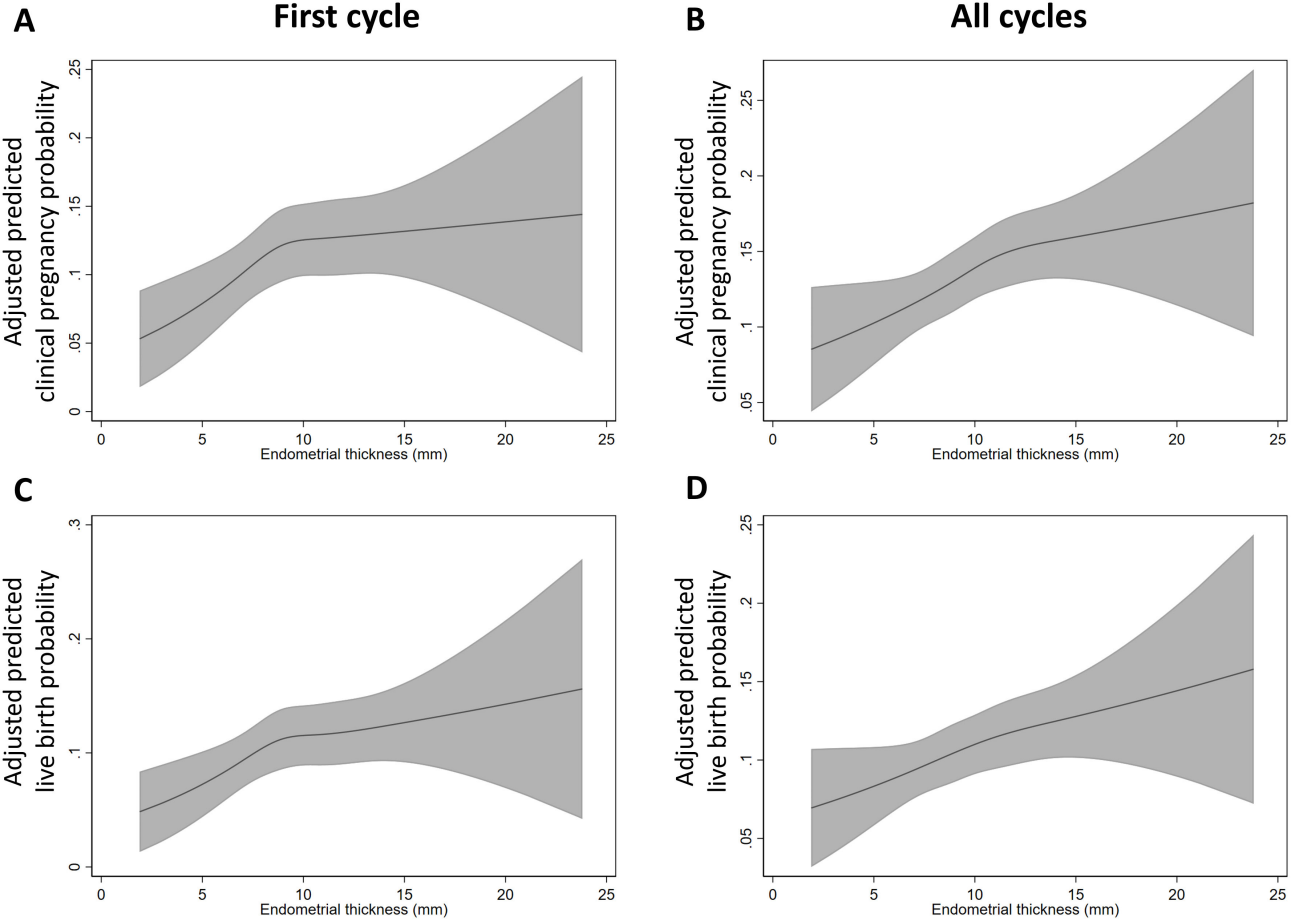
Predicted probability of clinical pregnancy (**(A)**, first cycle; **(B)** all cycles) and live birth (**(C)**, first cycle; **(D)** all cycles) for continuous endometrial thickness on trigger day. Shaded areas are 95% CI.

The restricted cubic spline analysis showed an approximately linear relationship between EMT and the probability of clinical pregnancy and live birth in the all cycles analysis (**Figures 3B, D**). In contrast, the first cycle analyses revealed a non-linear relationship, with an inflection point around 10 mm (**Figures 3A, C**). Before this point, the slope was steeper, indicating that increases in EMT had a greater impact on clinical pregnancy and live birth rates. Beyond 10 mm, the slope became less steep, suggesting a diminishing effect of EMT on these outcomes.

### Sensitivity analysis: generalized estimation equation and logistic regression

There were 2279 IUI cycles had an EMT<8mm on trigger day, 7126 IUI cycles had an EMT of 8-11mm, and 3698 IUI cycles had an EMT>11mm when using wider grouping intervals for thin and thick EMT groups. Characteristics of the EMT groups are shown in **Supplementary Table 3**.

In line with the results of the main analyses, across all cycles, compared with the medium EMT group (16.3%), the chance of clinical pregnancy was lower in the thin EMT group (13.3%; aOR 0.82, 95% CI 0.73–0.92) and higher in the thick EMT group (19.0%; aOR 1.16, 95% CI 1.06–1.26). Similarly, live birth rates were lower in the thin EMT group (10.7%) and higher in the thick EMT group (15.6%), compared to the medium EMT group (13.3%)—aOR 0.82 (95% CI 0.72–0.94) for thin EMT and aOR 1.14 (95% CI 1.04–1.26) for thick EMT. The associations between EMT and fertility outcomes were consistent when analyzing first cycles only. (**Supplementary Table 4**)

In the treatment protocol subgroup analysis, the trends in clinical pregnancy and live birth rates across the thin, medium, and thick EMT groups were consistent between natural cycles and Letrozole with or without hMG cycles. (**Supplementary Table 5**)

## Discussion

### Key findings

In this large cohort study, we found that EMT is significantly positively associated with the chance of both clinical pregnancy and live birth in IUI cycles.

### Comparisons of results between ours and previous studies

Most previous researches found no evidence for an association between EMT and pregnancy chances, including a recent meta-analysis (5).

Only a few studies observed positive association. In 1993, Dickey et al. (14) prospectively studied 474 cycles and reported increasing ongoing pregnancy rates with thicker EMT intervals in univariate analysis without confounding addressing. About 20 years later, Wolff et al. (15) retrospectively studied 2929 first IUI cycles and found clinical pregnancy rates increased significantly with increasing EMT. However, there was no definition of EMT groups in this study, and only ≥14 mm follicles were adjusted. Recently, Tang et al. (16) retrospectively studied 1464 IUI cycles, and found the <8mm EMT group was associated with a significantly higher risk of not achieving clinical pregnancy than the 8–13 mm group, but no improved pregnancy rate was found for the >13mm group. This study neither studied live birth, nor considered follicles.

Most previous studies on this topic share common limitations. First, the clinical pregnancy rate of a single IUI is typically around 15%, requiring a large sample size to achieve sufficient statistical power. However, the available literature predominantly consists of studies with small (hundreds) to medium (1000–2000) sample sizes. Moreover, the subset of cycles with thin EMT is even smaller, further reducing the ability to detect differences across various EMT groups. Second, endometrium and embryo are the two most crucial factors for achieving a clinical pregnancy. The number and size of follicles at trigger are significant confounders when studying the independent effect of EMT on clinical pregnancy. Previous research has shown that all follicles that ≥10mm at trigger independently contribute to clinical pregnancy in IUI, with their contribution varying significantly according to the number and size of the follicles (17). However, most previous studies on this topic failed to adequately adjust for these variables, or did not account for them at all, compromising their results’ reliability. In fact, cycles with more and larger follicles are typically associated with a thicker endometrium due to higher estrogen, especially in gonadotrophin-stimulated cycles. Therefore, ignoring follicle factors is prone to introduce serious biases. Third, when modeling the effect of EMT as a continuous variable, the effect of EMT on clinical pregnancy may not be consistent per mm. For example, when reaching a certain thickness, an additional 1 mm increase in EMT is unlikely to have the same effect on clinical pregnancy as it would when the endometrium is thin. Despite this, many previous studies assumed a linear relationship between EMT and pregnancy outcomes, which oversimplifies the potentially complex relationship. Fourth, the effect of EMT on other fertility outcomes of clinical interests following IUI, such as miscarriage, ectopic pregnancy and multiple pregnancy, was rarely studied.

In light of these limitations, our study included more than 13000 IUI cycles, representing the largest sample size reported on this topic to date. We exhaustively adjusted for the number and size intervals of all follicles ≥10 mm at trigger. We did not assume a linear relationship when exploring the association between EMT and pregnancy outcomes. Moreover, to enhance clinical relevance and facilitate clinical application, we classified the EMT into 3 ordered categories (thin, medium, and thick), and studied a range of fertility outcomes beyond clinical pregnancy.

### Interpretation, implications, and future research directions

The results of this study highlight the potential value of treatment strategies aimed at improving EMT, particularly for women with thin endometrium. As the results of the all cycle analysis shows, if the EMT could be improved from <7 mm to >14 mm, there might be a 53.6% (5.9/11.0) increase in live birth odds for a single IUI, which is of important clinical significance. Even if being improved from 7–14 mm to >14 mm, a 25.2% (3.4/13.5) increase in live birth odds for a single IUI is also of clinical value.

The utility of estrogen supplementation in IUI cycles remains largely unstudied. A small sample size study showed estrogen supplementation could improve EMT, but whether this improvement could translate into improved IUI outcomes still needs further well designed and powered research to clarify (11).

Mao et al. conducted a single center randomized controlled trial among infertile women with unresponsive thin (<7 mm) endometrium, and results showed that intrauterine infusion of granulocyte macrophage colony-stimulating factor (GM-CSF) could effectively increase endometrial thickness and improved the clinical pregnancy rate in frozen-thawed embryo transfer cycles (24). Further mechanism research using rat models reveled that thin endometrium was associated with significantly decreased molecular markers of endometrial receptivity, including homeobox A10 gene, leukemia inhibitory factor, integrin subunit β3, matrix metalloproteinase 9, and calcitonin. GM-CSF treatment could effectively upregulate the expression of these markers through the mitogen-activated protein kinase/extracellular signal-regulated kinase pathway, and increase endometrial thickness as well as number of offspring (25). These findings were in line with our results. Continued research and development in this area hold the potential to enhance fertility outcomes.

Our results showed a significantly increased multiple gestation risk for women with a >14 mm EMT, which was in accordance with their significantly higher clinical and live birth odds, indicating thicker endometrium is associated with better endometrial receptivity. On the other hand, this emphasizes the need to pay attention to evaluating the multiple gestation risk of a IUI cycle with both multiple >12 mm follicles and a thick EMT (17). However, from a treatment perspective, since modifying the endometrium remains challenging for women with unresponsive and repeated thin endometrium, optimizing modifiable factors - such as the combination of follicle number and size – could hold promise. Adjusting controlled ovary stimulation to appropriately increase the number of follicles of a certain size during IUI may enhance fertility success chance while limiting the risk of multiple gestation (17). This potential “follicular strategy for thin endometrium” merits further study.

EMT can also serve as a prognostic factor, alongside other critical variables (17), to predict the likelihood of success in IUI. This is valuable for informing patients and setting realistic expectations. However, we believe that thin EMT should not be used as an absolute medical indication to cancel IUI cycles. Our data shows, even in the thin EMT group, the live birth rate surpasses 10%. Therefore, the choice of cancelling an IUI cycle with thin EMT should be offered to fully informed patients based on their individual circumstances, including a cost-effective analysis weighing the sunk cost (e.g.: a month’s time, multiple hospital visits, blood hormone tests, vaginal ultrasounds, medications, and injections) and the future cost (the expense of the final IUI operation), given that the sunk cost may equal or even exceed the future cost at a late follicular stage near trigger during an IUI cycle.

### Strengths and limitations

The current study used a large-scale IUI dataset, which includes detailed records of both EMT and the number and size of all follicles ≥10 mm at trigger. This comprehensive data enabled us to examine the effect of EMT on various fertility outcomes while rigorously accounting for critical confounding.

Given the relatively low clinical pregnancy rate per single cycle, IUI is typically recommended for 3–6 attempts before transitioning to IVF. In this study, we adopted a multi-cycle analysis strategy using Generalized Estimating Equation Regression Models to account for the correlation between cycles from the same women. Additionally, we conduct separate analyses using data from only the first cycle of each participant. The consistent conclusions from both approaches further validated our findings.

In the first cycle analysis, we applied both Propensity Score Matching Analysis and Inverse Propensity Score Weighted Regression Adjustment Analysis. Propensity Score Matching has distinct advantages, such as being more straightforward and easier to interpret; however, it does discard data that do not match closely between treatment and control groups, which can lead to selection bias and reduced generalizability (26). This also contributes to a reduction in statistical power – as seen in this study, since the medium EMT group is much larger than the thin or thick EMT groups, a majority of the patients in the medium EMT group were discarded in Propensity Score Matching. Considering this, we also conducted an Inverse Propensity Score Weighted Regression Adjustment Analysis, a doubly robust method that also utilizes propensity scores and makes full use of all patients’ data. The results of the two sets of analyses concurred and are complementary.

To explore the varying impact of different EMT levels on fertility success rates, we estimated the predicted probabilities of primary outcomes for various EMT values at trigger, adjusting for potential confounders. These estimates provided a clear visualization of the relationship between EMT and fertility outcomes, offering added value for clinical decision-making and patient counseling.

As for the categorized EMT analyses, both a common practice cut-off (< 7.0 mm, 7.0-14.0 mm, > 14.0 mm) (19, 20) and a wider cut-off (< 8.0mm, 8.0-11.0 mm, > 11.0 mm) were used in the main and sensitivity analyses respectively, to assess the stability and reliability of the computed associations of EMT and fertility outcomes. The results from both sets of analyses were consistent, which reinforce the robustness of the primary findings and mitigate concerns regarding the specific cut-off values chosen.

The current study also has limitations. One limitation is the potential of residual confounding due to its retrospective design, despite our efforts to statistically address numerous confounders. Endometrial pattern was not routinely measured in our center, thus the current study was unable to address this potential confounder. Also, inter-observer and intra-observer variability in EMT measurement might introduce biases. Well-designed and adequately powered randomized controlled trials with appropriate interventions to improving EMT are needed to test our findings and the causality between EMT and fertility outcomes. Second, although this study is based on a large sample size, it may still lack sufficient power to yield robust conclusions for events with extremely low rates, such as ectopic pregnancy. Similarly, the number of first IUI cycles was relatively limited, which may have constrained the ability to detect statistically significant differences between the medium and thick EMT groups, and compromised the smoothness of the restricted cubic spline curve and the assessment of linearity. Third, the smaller number of cycles with very thin or very thick EMT resulted in wider 95% CIs for the predicted probabilities in these groups compared to those with medium EMT, reflecting greater variability in the estimates.

## Conclusions

In natural or Letrozole with or without hMG stimulated IUI cycles, EMT has an independent and significant positive association with the chance of live birth. As such, EMT on the trigger day could serve as a prognostic factor for IUI success. Treatment strategies that improve EMT in women with a thin endometrium may enhance IUI outcomes and warrant further research.

## Data Availability

The data analyzed in this study is subject to the following licenses/restrictions: Summary data of individual patients are available in the manuscript or additional files. Individual level data underlying this article cannot be shared publicly to protect the privacy of individuals included in the study. Requests to access these datasets should be directed to ocyte@qq.com.
