# Supplementary material for: Is endometrial thickness associated with fertility outcomes in intrauterine insemination? a cohort study
Source: Front Endocrinol (Lausanne). 2026 Jan 9;16:1705695. doi: 10.3389/fendo.2025.1705695 (PMC12827171; doi:10.3389/fendo.2025.1705695)
Supplement: Supplementary file 1 [file Table1.docx]

**Supplementary Table 1 First cycle analysis: Covariate balance summary in comparing thin endometrium (<7mm) to medium endometrium (7-14mm) in the first cycles** **using Inverse Propensity Score Weighted Regression Adjustment.**

| **Variable** | **Standardized difference** | |  | **Variance ratio** | |
| --- | --- | --- | --- | --- | --- |
|  | **Unweighted** | **Weighted** |  | **Unweighted** | **Weighted** |
| **Maternal characteristics, %** |  |  |  |  |  |
| Female age |  |  |  |  |  |
| <30 | Reference | Reference |  | Reference | Reference |
| 30-34 | -0.0274343 | -0.0328376 |  | 0.9956981 | 0.9924932 |
| 35-37 | 0.025211 | -0.0242087 |  | 1.055365 | 0.9489326 |
| 38-40 | 0.1084974 | 0.0258962 |  | 1.469479 | 1.103068 |
| 41-43 | 0.0222349 | 0.0012153 |  | 1.182475 | 1.009415 |
| ≥44 | 0.0720047 | 0.016098 |  | 2.017614 | 1.186171 |
| Body mass index |  |  |  |  |  |
| <18.5 | Reference | Reference |  | Reference | Reference |
| 18.5-23.9 | 0.0280399 | 0.0309061 |  | 1.068113 | 1.072661 |
| 24-28 | 0.0743094 | -0.0408452 |  | 1.124442 | 0.931775 |
| >28 | -0.0146087 | 0.0119456 |  | 0.9504488 | 1.042571 |
| Infertility duration |  |  |  |  |  |
| <1 | Reference | Reference |  | Reference | Reference |
| 1-2 | 0.0872245 | -0.007494 |  | 1.032243 | 0.9967828 |
| 3-4 | -0.0503869 | 0.0200642 |  | 0.9536275 | 1.018184 |
| 5-6 | -0.1058143 | 0.0042205 |  | 0.7527912 | 1.010328 |
| ≥7 | -0.0827819 | 0.0233052 |  | 0.7252275 | 1.084092 |
| Gravidity |  |  |  |  |  |
| 0 | Reference | Reference |  | Reference | Reference |
| 1 | 0.1249826 | 0.0294815 |  | 1.189478 | 1.044772 |
| ≥2 | 0.2699221 | -0.003756 |  | 1.64368 | 0.9914729 |
| Previous full-term birth |  |  |  |  |  |
| 0 | Reference | Reference |  | Reference | Reference |
| 1 | 0.0674989 | 0.0567678 |  | 1.239453 | 1.195742 |
| 2 | 0.0176495 | -0.0178131 |  | 1.286587 | 0.7506955 |
| Previous miscarriage |  |  |  |  |  |
| 0 | Reference | Reference |  | Reference | Reference |
| 1 | 0.1531741 | 0.0221721 |  | 1.227846 | 1.03344 |
| ≥2 | 0.2697885 | -0.023452 |  | 1.773595 | 0.9390295 |
| Previous ectopic pregnancy |  |  |  |  |  |
| 0 | Reference | Reference |  | Reference | Reference |
| ≥1 | 0.0795114 | -0.0089323 |  | 1.989582 | 0.9136467 |
| Smoking history |  |  |  |  |  |
| Yes | 0.0259082 | -0.0177641 |  | 2.073421 | 0.4993851 |
| No | Reference | Reference |  | Reference | Reference |
| PCOS |  |  |  |  |  |
| Yes | 0.1224285 | -0.0053847 |  | 1.235809 | 0.9897686 |
| No | Reference | Reference |  | Reference | Reference |
| **Paternal characteristics, %** |  |  |  |  |  |
| Paternal age |  |  |  |  |  |
| <30 | Reference | Reference |  | Reference | Reference |
| 30-39 | -0.0868599 | -0.0231335 |  | 1.058402 | 1.016075 |
| 40-49 | 0.1253725 | -0.0183825 |  | 1.362818 | 0.9502474 |
| ≥50 | 0.1447682 | -0.0129256 |  | 3.697518 | 0.8592863 |
| Mild male factor |  |  |  |  |  |
| Yes | -0.086172 | 0.026371 |  | 0.9368859 | 1.017616 |
| No | Reference | Reference |  | Reference | Reference |
| **IUI characteristics, %** |  |  |  |  |  |
| Follicle number on trigger day |  |  |  |  |  |
| <10 mm |  |  |  |  |  |
| 0 | Reference | Reference |  | Reference | Reference |
| 1 | 0.0805093 | -0.0131694 |  | 1.291706 | 0.9555748 |
| ≥2 | 0.2261366 | 0.0080243 |  | 1.241667 | 1.009504 |
| 10-12 mm |  |  |  |  |  |
| 0 | Reference | Reference |  | Reference | Reference |
| 1 | 0.0844908 | 0.009645 |  | 1.194951 | 1.021606 |
| ≥2 | 0.0258012 | 0.0467403 |  | 1.093518 | 1.166906 |
| 12-14 mm |  |  |  |  |  |
| 0 | Reference | Reference |  | Reference | Reference |
| 1 | 0.0468447 | -0.0248403 |  | 1.099459 | 0.9487166 |
| ≥2 | -0.0718722 | -0.0142896 |  | 0.7541368 | 0.9483442 |
| 14-16 mm |  |  |  |  |  |
| 0 | Reference | Reference |  | Reference | Reference |
| 1 | -0.0530862 | 0.0050433 |  | 0.9049182 | 1.009214 |
| ≥2 | -0.1358325 | 0.0106882 |  | 0.5623988 | 1.038813 |
| 16-18 mm |  |  |  |  |  |
| 0 | Reference | Reference |  | Reference | Reference |
| 1 | -0.0874039 | -0.0227247 |  | 0.8943269 | 0.9725531 |
| ≥2 | -0.2049768 | 0.0562824 |  | 0.3924439 | 1.202389 |
| 18-20 mm |  |  |  |  |  |
| 0 | Reference | Reference |  | Reference | Reference |
| 1 | -0.0098142 | 0.0209815 |  | 0.9976346 | 1.008121 |
| ≥2 | -0.1248627 | 0.0110551 |  | 0.62897 | 1.036108 |
| >20 mm |  |  |  |  |  |
| 0 | Reference | Reference |  | Reference | Reference |
| 1 | 0.0653273 | 0.0249835 |  | 1.029776 | 1.011408 |
| ≥2 | -0.0426701 | -0.0117065 |  | 0.8131002 | 0.9463749 |
| Year of treatment |  |  |  |  |  |
| 2007-2009 | Reference | Reference |  | Reference | Reference |
| 2010-2012 | -0.065693 | -0.0271768 |  | 0.8185835 | 0.9224198 |
| 2013-2015 | -0.134891 | 0.0199793 |  | 0.8554555 | 1.020024 |
| 2016-2018 | 0.0738016 | 0.0187217 |  | 1.030558 | 1.008082 |
| 2019-2021 | 0.1031707 | -0.0261061 |  | 1.134836 | 0.9648703 |

Note: PCOS, polycystic ovary syndrome.

**Supplementary Table 2 First cycle analysis: Covariate balance summary in comparing thick endometrium (>14mm) to medium endometrium (7-14mm) in the first cycles** **using Inverse Propensity Score Weighted Regression Adjustment.**

| **Variable** | **Standardized difference** | |  | **Variance ratio** | |
| --- | --- | --- | --- | --- | --- |
|  | **Unweighted** | **Weighted** |  | **Unweighted** | **Weighted** |
| **Maternal characteristics, %** |  |  |  |  |  |
| Female age |  |  |  |  |  |
| <30 | Reference | Reference |  | Reference | Reference |
| 30-34 | 0.0459735 | -0.0087229 |  | 1.009201 | 0.9982968 |
| 35-37 | -0.0896148 | 0.0367933 |  | 0.8138162 | 1.078858 |
| 38-40 | -0.0392395 | 0.0486584 |  | 0.849779 | 1.202557 |
| 41-43 | -0.0463718 | -0.0557582 |  | 0.669835 | 0.6044197 |
| ≥44 | 0.0057569 | 0.027523 |  | 1.070248 | 1.341964 |
| Body mass index |  |  |  |  |  |
| <18.5 | Reference | Reference |  | Reference | Reference |
| 18.5-23.9 | -0.099057 | -0.0813051 |  | 0.7736223 | 0.8099402 |
| 24-28 | 0.0165443 | 0.015255 |  | 1.030177 | 1.025423 |
| >28 | 0.0530671 | -0.0258131 |  | 1.19679 | 0.9105935 |
| Infertility duration |  |  |  |  |  |
| <1 | Reference | Reference |  | Reference | Reference |
| 1-2 | -0.0254255 | -0.0019325 |  | 0.9907448 | 0.9991511 |
| 3-4 | 0.0341598 | -0.0548274 |  | 1.032789 | 0.94789 |
| 5-6 | 0.1060965 | 0.029473 |  | 1.262065 | 1.070385 |
| ≥7 | 0.0297356 | 0.0368634 |  | 1.109009 | 1.131648 |
| Gravidity |  |  |  |  |  |
| 0 | Reference | Reference |  | Reference | Reference |
| 1 | -0.1145875 | 0.0646496 |  | 0.8190237 | 1.100571 |
| ≥2 | -0.2250245 | -0.0103929 |  | 0.5005341 | 0.9748421 |
| Previous full-term birth |  |  |  |  |  |
| 0 | Reference | Reference |  | Reference | Reference |
| 1 | -0.0953169 | -0.0030681 |  | 0.696225 | 0.9894968 |
| 2 | -0.0293676 | -0.0235483 |  | 0.6041345 | 0.6713629 |
| Previous miscarriage |  |  |  |  |  |
| 0 | Reference | Reference |  | Reference | Reference |
| 1 | -0.1614192 | 0.086152 |  | 0.7440876 | 1.133129 |
| ≥2 | -0.2280324 | -0.0358181 |  | 0.4343631 | 0.9008835 |
| Previous ectopic pregnancy |  |  |  |  |  |
| 0 | Reference | Reference |  | Reference | Reference |
| ≥1 | -0.0867879 | 0.0529742 |  | 0.2839877 | 1.631191 |
| Smoking history |  |  |  |  |  |
| Yes | / | / |  | / | / |
| No | Reference | Reference |  | Reference | Reference |
| PCOS |  |  |  |  |  |
| Yes | -0.0699068 | 0.0338343 |  | 0.8667772 | 1.065932 |
| No | Reference | Reference |  | Reference | Reference |
| **Paternal characteristics, %** |  |  |  |  |  |
| Paternal age |  |  |  |  |  |
| <30 | Reference | Reference |  | Reference | Reference |
| 30-39 | 0.0104113 | -0.0074903 |  | 0.9947539 | 1.00543 |
| 40-49 | 0.0089234 | 0.0622506 |  | 1.027452 | 1.176227 |
| ≥50 | -0.0569902 | -0.0537752 |  | 0.4082987 | 0.4274218 |
| Mild male factor |  |  |  |  |  |
| Yes | 0.0543314 | -0.0250307 |  | 1.036697 | 0.9826574 |
| No | Reference | Reference |  | Reference | Reference |
| **IUI characteristics, %** |  |  |  |  |  |
| Follicle number on trigger day |  |  |  |  |  |
| <10 mm |  |  |  |  |  |
| 0 | Reference | Reference |  | Reference | Reference |
| 1 | -0.1132868 | 0.0136242 |  | 0.6375842 | 1.047974 |
| ≥2 | -0.0099768 | -0.051717 |  | 0.9900017 | 0.9342371 |
| 10-12 mm |  |  |  |  |  |
| 0 | Reference | Reference |  | Reference | Reference |
| 1 | -0.0310109 | -0.0095563 |  | 0.9322204 | 0.9782811 |
| ≥2 | 0.0178248 | -0.0433757 |  | 1.065631 | 0.8525114 |
| 12-14 mm |  |  |  |  |  |
| 0 | Reference | Reference |  | Reference | Reference |
| 1 | 0.0696604 | 0.0178423 |  | 1.14787 | 1.036861 |
| ≥2 | 0.0699981 | -0.0181746 |  | 1.265983 | 0.9359193 |
| 14-16 mm |  |  |  |  |  |
| 0 | Reference | Reference |  | Reference | Reference |
| 1 | 0.0282602 | 0.0170012 |  | 1.053659 | 1.030648 |
| ≥2 | 0.191041 | 0.0329725 |  | 1.744387 | 1.115138 |
| 16-18 mm |  |  |  |  |  |
| 0 | Reference | Reference |  | Reference | Reference |
| 1 | 0.1207944 | 0.0019135 |  | 1.134032 | 1.002215 |
| ≥2 | 0.0979134 | -0.0109375 |  | 1.351232 | 0.9637018 |
| 18-20 mm |  |  |  |  |  |
| 0 | Reference | Reference |  | Reference | Reference |
| 1 | -0.110703 | -0.0510393 |  | 0.9461779 | 0.9762219 |
| ≥2 | 0.1473875 | -0.0104491 |  | 1.502718 | 0.9674944 |
| >20 mm |  |  |  |  |  |
| 0 | Reference | Reference |  | Reference | Reference |
| 1 | -0.0259734 | 0.0574599 |  | 0.9890373 | 1.025237 |
| ≥2 | 0.0617205 | -0.0219054 |  | 1.302919 | 0.9023876 |
| Year of treatment |  |  |  |  |  |
| 2007-2009 | Reference | Reference |  | Reference | Reference |
| 2010-2012 | 0.2017378 | -0.0030774 |  | 1.608403 | 0.9914554 |
| 2013-2015 | -0.0309424 | 0.0005479 |  | 0.9709107 | 1.000549 |
| 2016-2018 | -0.1728032 | 0.017748 |  | 0.8961475 | 1.008254 |
| 2019-2021 | -0.0526294 | -0.02575 |  | 0.9296688 | 0.9645895 |

Note: PCOS, polycystic ovary syndrome. Smoking was dropped because of collinearity.

**Supplementary Table 3 All cycle analysis: Characteristics of the three endometrial thickness groups in all cycles using wider grouping cut-offs.**

| **Variable** | **Thin group**  **（＜8 mm）** | **Medium group**  **（8-11 mm）** | **Thick group**  **（＞11 mm）** | ***P*** |
| --- | --- | --- | --- | --- |
| **Total cycles, n** | 2279 | 7126 | 3698 |  |
| **Maternal characteristics, %** |  |  |  |  |
| Female age |  |  |  | **<0.001** |
| <30 | 29.2 | 31.7 | 33.2 |  |
| 30-34 | 44.6 | 46.0 | 46.6 |  |
| 35-37 | 15.7 | 14.4 | 13.4 |  |
| 38-40 | 7.8 | 5.6 | 4.8 |  |
| 41-43 | 1.8 | 1.5 | 1.5 |  |
| ≥44 | 1.0 | 0.8 | 0.5 |  |
| Body mass index |  |  |  | 0.257 |
| <18.5 | 12.9 | 12.2 | 11.3 |  |
| 18.5-23.9 | 62.5 | 63.8 | 63.1 |  |
| 24-28 | 18.6 | 17.6 | 18.6 |  |
| >28 | 6.1 | 6.4 | 7.0 |  |
| Infertility duration |  |  |  | **0.001** |
| <1 | 15.3 | 13.5 | 12.2 |  |
| 1-2 | 40.3 | 38.9 | 38.4 |  |
| 3-4 | 28.3 | 29.3 | 29.7 |  |
| 5-6 | 10.2 | 11.9 | 12.1 |  |
| ≥7 | 5.9 | 6.4 | 7.7 |  |
| Gravidity |  |  |  | **<0.001** |
| 0 | 58.4 | 69.2 | 76.3 |  |
| 1 | 22.8 | 19.9 | 15.8 |  |
| ≥2 | 18.8 | 10.9 | 7.9 |  |
| Previous preterm birth |  |  |  | 0.945 |
| 0 | 99.7 | 99.8 | 99.7 |  |
| ≥1 | 0.3 | 0.2 | 0.3 |  |
| Previous full-term birth |  |  |  | **0.009** |
| 0 | 91.3 | 93.1 | 93.6 |  |
| 1 | 8.3 | 6.5 | 6.1 |  |
| 2 | 0.4 | 0.4 | 0.3 |  |
| Previous miscarriage |  |  |  | **<0.001** |
| 0 | 59.7 | 71.1 | 79.0 |  |
| 1 | 24.2 | 19.9 | 15.0 |  |
| ≥2 | 16.1 | 8.9 | 6.0 |  |
| Previous ectopic pregnancy |  |  |  | **0.023** |
| 0 | 98.7 | 99.0 | 99.4 |  |
| ≥1 | 1.3 | 1.0 | 0.6 |  |
| Smoking history |  |  |  | 0.077 |
| Yes | 0.2 | 0.1 | 0.0 |  |
| No | 99.8 | 99.9 | 100.0 |  |
| PCOS |  |  |  | 0.257 |
| Yes | 15.4 | 15.5 | 14.4 |  |
| No | 84.6 | 84.5 | 85.6 |  |
| **Paternal characteristics, %** |  |  |  |  |
| Paternal age |  |  |  | 0.110 |
| <30 | 20.5 | 21.4 | 21.5 |  |
| 30-39 | 67.0 | 68.4 | 68.0 |  |
| 40-49 | 11.6 | 9.5 | 9.8 |  |
| ≥50 | 0.9 | 0.7 | 0.6 |  |
| Mild male factor |  |  |  | 0.117 |
| Yes | 32.9 | 34.5 | 35.5 |  |
| No | 67.1 | 65.5 | 64.5 |  |
| **IUI characteristics, %** |  |  |  |  |
| Treatment protocol |  |  |  | 0.120 |
| Letrozole stimulation | 94.6 | 94.6 | 93.7 |  |
| Natural cycle | 5.4 | 5.4 | 6.3 |  |
| Follicle number on trigger day |  |  |  |  |
| <10 mm |  |  |  | **<0.001** |
| 0 | 60.7 | 69.9 | 73.6 |  |
| 1 | 8.4 | 6.8 | 5.5 |  |
| ≥2 | 30.8 | 23.3 | 21.0 |  |
| 10-12 mm |  |  |  | 0.288 |
| 0 | 80.8 | 81.0 | 81.3 |  |
| 1 | 13.3 | 12.3 | 11.8 |  |
| ≥2 | 5.8 | 6.7 | 6.8 |  |
| 12-14 mm |  |  |  | **0.023** |
| 0 | 80.8 | 80.6 | 79.6 |  |
| 1 | 14.5 | 13.4 | 13.8 |  |
| ≥2 | 4.7 | 5.9 | 6.7 |  |
| 14-16 mm |  |  |  | **<0.001** |
| 0 | 81.1 | 78.7 | 74.1 |  |
| 1 | 15.0 | 16.1 | 17.2 |  |
| ≥2 | 3.9 | 5.2 | 8.7 |  |
| 16-18 mm |  |  |  | **<0.001** |
| 0 | 73.6 | 70.6 | 65.4 |  |
| 1 | 22.4 | 23.1 | 26.1 |  |
| ≥2 | 4.0 | 6.3 | 8.5 |  |
| 18-20 mm |  |  |  | **<0.001** |
| 0 | 55.2 | 52.8 | 51.9 |  |
| 1 | 39.7 | 40.5 | 38.2 |  |
| ≥2 | 5.1 | 6.7 | 9.8 |  |
| >20 mm |  |  |  | **0.042** |
| 0 | 57.1 | 57.0 | 57.2 |  |
| 1 | 39.5 | 39.2 | 38.0 |  |
| ≥2 | 3.4 | 3.8 | 4.8 |  |
| Year of treatment |  |  |  | **<0.001** |
| 2007-2009 | 2.1 | 2.1 | 3.0 |  |
| 2010-2012 | 8.2 | 8.2 | 11.5 |  |
| 2013-2015 | 24.4 | 27.6 | 27.2 |  |
| 2016-2018 | 40.3 | 40.2 | 39.4 |  |
| 2019-2021 | 25.1 | 21.9 | 18.9 |  |

Note: PCOS, polycystic ovary syndrome; Comparisons were made using chi-square test or Fisher’s exact test as appropriate. Bold indicates significant *P*-values.

| **Supplementary Table 4 Fertility outcomes of the three endometrial thickness groups using wider grouping cut-offs in first and in all cycles.** | | | | | | | |
| --- | --- | --- | --- | --- | --- | --- | --- |
| **Outcome** | **First cycle**^a^ | | |  | **All cycles**^b^ | | |
|  | **Rate,%**  **(n=7609)** | **Crude OR**  **(95% CI)** | **Adjusted OR**  **(95% CI)** |  | **Rate,%**  **(n= 13103)** | **Crude OR**  **(95% CI)** | **Adjusted OR**  **(95% CI)** |
| Biochemical pregnancy loss |  |  |  |  |  |  |  |
| ＜8 mm | 0.6 | 0.93 (0.42-2.05) | 0.83 (0.37-1.87) |  | 0.7 | 1.30 (0.71-2.38) | 1.26 (0.68-2.37) |
| 8-11 mm | 0.6 | 1 (Reference) | 1 (Reference) |  | 0.5 | 1 (Reference) | 1 (Reference) |
| ＞11 mm | 0.5 | 0.82 (0.41-1.67) | 0.89 (0.43-1.82) |  | 0.5 | 1.02 (0.57-1.81) | 1.06 (0.59-1.93) |
| Clinical pregnancy |  |  |  |  |  |  |  |
| ＜8 mm | 14.3 | **0.74 (0.62-0.88)** | **0.74 (0.62-0.88)** |  | 13.3 | **0.82 (0.73-0.92)** | **0.82 (0.73-0.92)** |
| 8-11 mm | 18.4 | 1 (Reference) | 1 (Reference) |  | 16.3 | 1 (Reference) | 1 (Reference) |
| ＞11 mm | 20.4 | 1.14 (1.00-1.30) | 1.11 (0.97-1.27) |  | 19.0 | **1.17 (1.07-1.27)** | **1.16 (1.06-1.26)** |
| Multiple gestation |  |  |  |  |  |  |  |
| ＜8 mm | 1.5 | 0.77 (0.47-1.25) | 0.78 (0.47-1.28) |  | 1.2 | 0.74 (0.49-1.12) | 0.82 (0.54-1.25) |
| 8-11 mm | 2.0 | 1 (Reference) | 1 (Reference) |  | 1.7 | 1 (Reference) | 1 (Reference) |
| ＞11 mm | 2.6 | 1.31 (0.93-1.86) | 1.20 (0.83-1.72) |  | 2.4 | **1.45 (1.11-1.91)** | **1.31 (1.00-1.72)** |
| Ectopic pregnancy |  |  |  |  |  |  |  |
| ＜8 mm | 0.6 | 1.15 (0.51-2.60) | 1.04 (0.44-2.47) |  | 0.4 | 0.92 (0.45-1.86) | 0.90 (0.45-1.79) |
| 8-11 mm | 0.5 | 1 (Reference) | 1 (Reference) |  | 0.5 | 1 (Reference) | 1 (Reference) |
| ＞11 mm | 0.3 | 0.55 (0.22-1.38) | 0.58 (0.23-1.47) |  | 0.3 | 0.68 (0.35-1.31) | 0.71 (0.37-1.35) |
| Miscarriage |  |  |  |  |  |  |  |
| ＜8 mm | 2.5 | 0.85 (0.58-1.25) | 0.84 (0.57-1.25) |  | 2.3 | 0.89 (0.65-1.21) | 0.86 (0.63-1.18) |
| 8-11 mm | 2.9 | 1 (Reference) | 1 (Reference) |  | 2.6 | 1 (Reference) | 1 (Reference) |
| ＞11 mm | 3.4 | 1.16 (0.86-1.56) | 1.12 (0.83-1.53) |  | 3.3 | **1.28 (1.02-1.61)** | 1.24 (0.98-1.56) |
| Live birth |  |  |  |  |  |  |  |
| ＜8 mm | 11.4 | **0.72 (0.60-0.87)** | **0.72 (0.59-0.87)** |  | 10.7 | **0.80 (0.70-0.92)** | **0.82 (0.72-0.94)** |
| 8-11 mm | 15.2 | 1 (Reference) | 1 (Reference) |  | 13.3 | 1 (Reference) | 1 (Reference) |
| ＞11 mm | 17.0 | 1.14 (0.99-1.32) | 1.12 (0.96-1.29) |  | 15.6 | **1.17 (1.06-1.29)** | **1.14 (1.04-1.26)** |

Note: OR, odds ratio; CI, confidence interval. Adjusted ORs were adjusted for female age, paternal age, body mass index, infertility duration, gravidity, previous preterm birth, previous full-term birth, previous miscarriage, previous ectopic pregnancy, smoking, polycystic ovary syndrome, mild male factor, treatment protocol, year of treatment, and follicle number and diameter on trigger day; Bold indicates significant *P*-values.

^a^ Using Binary Logistic Regression Models.

^b^ Using Generalized Estimating Equations Models for correlated outcomes among all cycles of the same woman.

| **Supplementary Table 5 Primary fertility outcomes of the three endometrial thickness groups in treatment protocol subgroups in all cycles.** | | | | | | | | |
| --- | --- | --- | --- | --- | --- | --- | --- | --- |
| **Outcome** | **Natural cycle** | | |  | | **Letrozole (with or without HMG) cycle** | | |
|  | **Rate, % (n/N)** | **Crude OR (95% CI)** | **Adjusted OR^a^ (95% CI)** |  | **Rate, % (n/N)** | | **Crude OR (95% CI)** | **Adjusted OR^a^ (95% CI)** |
| Clinical pregnancy |  |  |  |  |  | |  |  |
| ＜8 mm | 7.3 (9/124) | 0.80 (0.37-1.73) | 0.77 (0.36-1.65) |  | 13.7 (295/2155) | | **0.79 (0.69-0.91)** | **0.80 (0.70-0.93)** |
| 8-11 mm | 8.9 (34/382) | 1 (Reference) | 1 (Reference) |  | 16.7 (1126/6744) | | 1 (Reference) | 1 (Reference) |
| ＞11 mm | 12.0 (28/233) | 1.40 (0.82-2.39) | 1.38 (0.80-2.38) |  | 19.5 (675/3465) | | **1.21 (1.09-1.34)** | **1.17 (1.05-1.30)** |
| Live birth |  |  |  |  |  | |  |  |
| ＜8 mm | 3.2 (4/124) | 0.52 (0.18-1.54) | 0.59 (0.19-1.80) |  | 11.1 (240/2155) | | **0.79 (0.68-0.91)** | **0.81 (0.69-0.94)** |
| 8-11 mm | 6.0 (23/382) | 1 (Reference) | 1 (Reference) |  | 13.8 (928/6744) | | 1 (Reference) | 1 (Reference) |
| ＞11 mm | 11.6 (27/233) | **2.05 (1.14-3.68)** | **1.90 (1.03-3.48)** |  | 15.9 (551/3465) | | **1.19 (1.06-1.33)** | **1.16 (1.03-1.30)** |

HMG=human menopausal gonadotropin; OR=odds ratio; CI=confidence interval.

^a^Analyzed using Generalized Estimating Equations Models for correlated outcomes among all cycles of the same woman, controlled for female age, body mass index, infertility duration, gravidity, previous full-term birth, previous miscarriage, previous ectopic pregnancy, smoking, PCOS, paternal age, mild male factor, year of treatment, and follicle number and diameter on trigger day (variables were excluded from the model in cases of complete separation); Bold indicates significant *P*-values.
